# A novel enzyme with antioxidant capacity produced by the ubiquitous skin colonizer *Propionibacterium acnes*

**DOI:** 10.1038/srep36412

**Published:** 2016-11-02

**Authors:** Maria Allhorn, Sabine Arve, Holger Brüggemann, Rolf Lood

**Affiliations:** 1 Department of Clinical Sciences Lund, Division of Infection Medicine, Biomedical Center B14, Lund University, Lund, Sweden; 2 Department of Biomedicine, Aarhus University, Aarhus, Denmark

## Abstract

The role of the skin microbiota in human health is poorly understood. Here, we identified and characterized a novel antioxidant enzyme produced by the skin microbiota, designated RoxP for radical oxygenase of *Propionibacterium acnes*. RoxP is uniquely produced by the predominant skin bacterium *P. acnes*, with no homologs in other bacteria; it is highly expressed and strongly secreted into culture supernatants. We show that RoxP binds heme, reduces free radicals, and can protect molecules from oxidation. Strikingly, RoxP is crucial for the survival of *P. acnes* in oxic conditions and for skin colonization of *P. acnes ex vivo*. Taken together, our study strongly suggests that RoxP facilitates *P. acnes*’ survival on human skin, and is an important beneficial factor for the host-commensal interaction. Thus, RoxP is the first described skin microbiota-derived mutualistic factor that potentially can be exploited for human skin protection.

*Propionibacterium acnes* is one of the most commonly isolated bacteria from human skin^1^,^2^,^3^. This Gram positive facultative anaerobe has been suggested to be involved in several skin disorders, acne vulgaris being the most notable^4^. Furthermore, *P. acnes* can adhere to and form biofilms on orthopedic implants^5^, and is therefore associated with infections of prosthetic devices^6^,^7^. However, *P. acnes* is an ubiquitous microorganism on skin, at certain parts outnumbering the aerobes 100-folds^8^; it can mono-colonize pilosebaceous units of human skin^9^ without causing any disease in most individuals. Therefore, it has been suggested that colonization by *P. acnes* might be beneficial for the human host^10^,^11^. In agreement with a beneficial function, absence of *P. acnes* has been associated with the development of psoriasis^12^, even though a putative protective role of *P. acnes* remains to be experimentally verified.

As a skin bacterium, *P. acnes* is constantly exposed to different environmental stresses, such as reactive oxygen species (ROS) produced from UV irradiated skin^13^, or from activated phagocytes^14^,^15^ and keratinocytes exposed to skin microbes^16^,^17^. Furthermore, *P. acnes* has to cope with the oxic environment present on the top layers of skin. *P. acnes* has evolved several defense mechanisms towards ROS. All strains are catalase positive^18^, and several strains also have a high superoxide dismutase activity^19^. However, these two enzymes do not have typical protein sorting motifs (*e.g.* no signal peptide or LPXTG motif) based on the sequenced genome of *P. acnes* strain KPA171202^20^,^21^, and are thus likely to be intracellular proteins. Recently, Holland *et al*. identified the secretome of *P. acnes* using mass spectrometry, and found that the most abundantly secreted protein was PPA1939, annotated as a hypothetical protein, with no homology to existing database entries^22^. We hypothesize that this protein is of importance for the bacterium to survive in its habitat.

Here, we have shown that PPA1939 is a secreted enzyme with antioxidant activity, unique to and favorable for *P. acnes* to survive in oxic conditions, with implications for maintaining redox homeostasis on human skin. Due to this activity, we tentatively name this protein RoxP – Radical oxygenase of *Propionibacterium acnes*.

## Results

### Phylogenetic relationship of RoxP (PPA1939)

RoxP (consisting of 161 amino acids) has so far only been annotated as a hypothetical protein, and has no conserved domains as judged from BLAST^23^ or pfam^24^ searches, besides an N-terminal signal peptide motif. A multi-genome comparison has revealed that this gene is present in the genomes of all *P. acnes* strains that have been sequenced so far (Supplementary Fig. 1). The only species, except *P. acnes*, that has a protein similar to RoxP is the closely related *Propionibacterium humerusii*. No other sequenced bacteria encode any proteins with sequence similarity to RoxP, according to a BLAST analysis.

**Figure 1:**
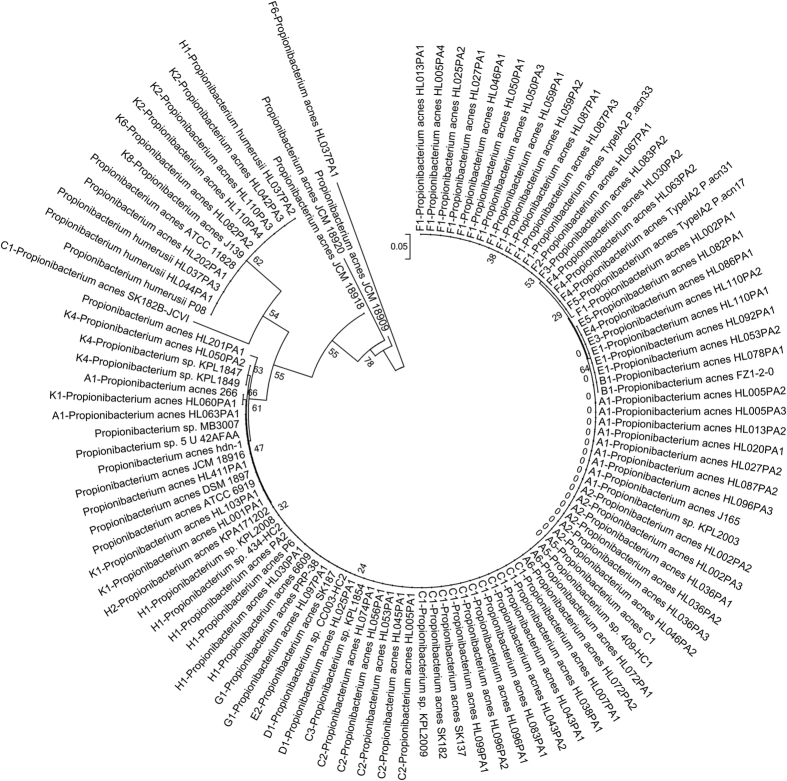
RoxP is highly conserved. The protein sequences of RoxP homologs were extracted from all available *P. acnes* genomes. The tree, generated by the minimum-evolution algorithm in MEGA version 5.2, illustrates that RoxP is highly conserved among the vast majority of *P. acnes* strains. Most strains (58) have an identical homolog, and 33 strains have highly similar homologs (99% identity). A subgroup of 11 RoxP homologs belonging to strains of *P. acnes* type II, type III, and *P. humerusii* are more distant (83% identity). Some genomes, such as JCM strains, encode a truncated RoxP. The bar represents the genetic distance.

To further study the conserved nature of *roxP*, a phylogenetic tree was constructed based on the amino acid sequences of RoxP homologs extracted from all publically available genomes of *P. acnes* and *P. humerusii* (Fig. 1, Supplementary Fig. 1). The phylogenetic tree reveals that RoxP is strikingly conserved. Most *P. acnes* strains have an identical RoxP, in particular strains of the *P. acnes* phylotype I, but also encompassing several strains of *P. acnes* type II. Using strain KPA171202 (type IB) as a reference strain, RoxP homologs in 58 strains are identical, while homologs in 33 other strains share a 99% sequence identity (160/161 amino acid identity). A distinct cluster (homologs with 83% identity) consists of 11 strains belonging to *P. humerusii* and some *P. acnes* type II. Strain SK182B harbors the most distantly related full-length RoxP homolog compared to the reference, sharing 81% amino acid identity. Noteworthy, four strains (three strains of different phylotypes are from the JCM collection) have a predicted truncated version of RoxP, due to a single base deletion (Supplementary Fig. 1). It cannot be ruled out that this represents sequencing errors, as the sequencing qualities of these genomes are low. No apparent discrepancy between phylotypes of *P. acnes* and the phylogenetic relationship of RoxP homologs could be found. In order to get a more complete picture, nine additional clinical isolates were screened for the presence of *roxP*, and sequenced (GenBank accession number JN051665-JN051673). All clinical isolates harbored a *roxP* that was identical or highly similar to *roxP* from previously sequenced genomes of *P. acnes* (83.2–100% amino acid identity), with seven strains being identical to KPA171202.

### Expression of *roxP*

**Figure 2:**
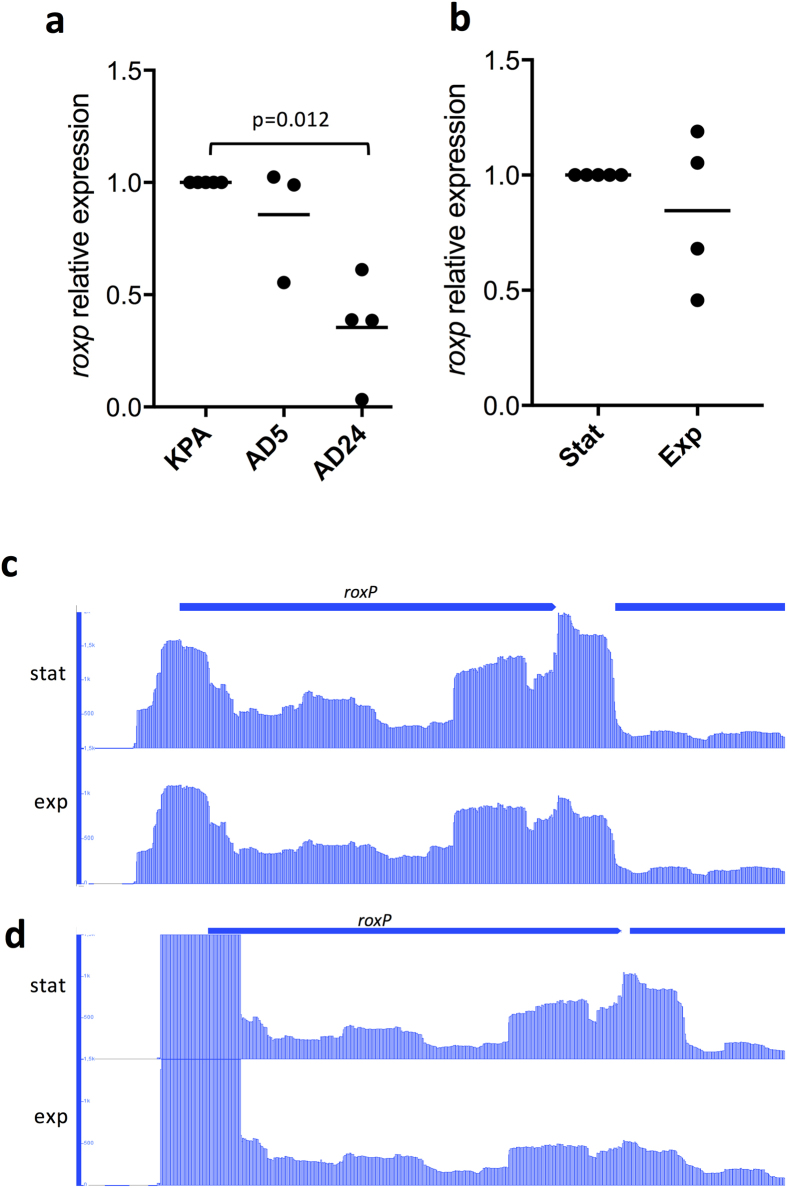
*roxP* is constitutively expressed in all strains. *roxP* expression was analyzed using qRT-PCR during exponential and stationary phase *P. acnes* strain KPA171202 (**a**) (n = 6). Differences in expression levels of *roxP* between individual isolates of *P. acnes* (KPA171202, AD5, AD24) was analyzed at exponential growth phase, normalized against *gapdh*, and presented as relative *roxP* expression with KPA171202 set as 1 (**b**) (n = 6). RNA-sequencing of *P. acnes* revealed that *roxP* is strongly expressed in both exponential and stationary growth phases in different phylotypes of *P. acnes* (**c**,**d**), as well as transcriptional elements controlling the expression of *roxP*.

A previous study, identifying all secreted proteins of *P. acnes*, has detected strong secretion of RoxP in all tested strains of the phylotypes IA, IB, II and III^22^. Even though *roxP* is present in all studied strains of *P. acnes*, the expression of the gene may vary between distinct isolates, as it has been indicated by a recent study measuring the surface proteome and secretome^25^. Here, we investigated the *roxP* expression in three *P. acnes* strains by means of qRT-PCR. *roxP* is expressed both during exponential and stationary phase (Fig. 2a). Furthermore, all tested isolates express similar levels of *roxP* compared to the reference strain KPA171202, except isolate AD24 (a type II strain) that expressed significantly lower amounts of *roxP* (Fig. 2b).

RNA-sequencing of *P. acnes* supported the qRT-PCR data, showing that *roxP* is strongly and constitutively expressed in strains belonging to two different phylotypes of *P. acnes* (Fig. 2c,d). This supports the notion that *roxP* is important throughout the entire *P. acnes* population. RNA-sequencing further revealed the transcriptional start site, the presence of a long 5′-untranslated region (UTR), and the possible promoter of *roxP* (Supplementary Fig. 2A). A phylogenetic analysis of the *roxP* upstream region, containing the transcriptional start site and the predicted promoter, in all *P. acnes* genomes revealed a highly conserved sequence (Supplementary Fig. 2B). The majority of strains (81%), including all type IA and IB strains and a few type II strains, possess a *roxP* upstream sequence that differs only in maximal 3 nucleotide positions. The remaining 18 strains belong to type IC, type II and type III as well as represent some strains of *P. humerusii*. In analogy with the RoxP phylogenetic analysis, strains of type II and *P. humerusii* cluster together, *i.e.* have the same *roxP* upstream region (Supplementary Fig. 2C).

### Purification of RoxP

**Figure 3:**
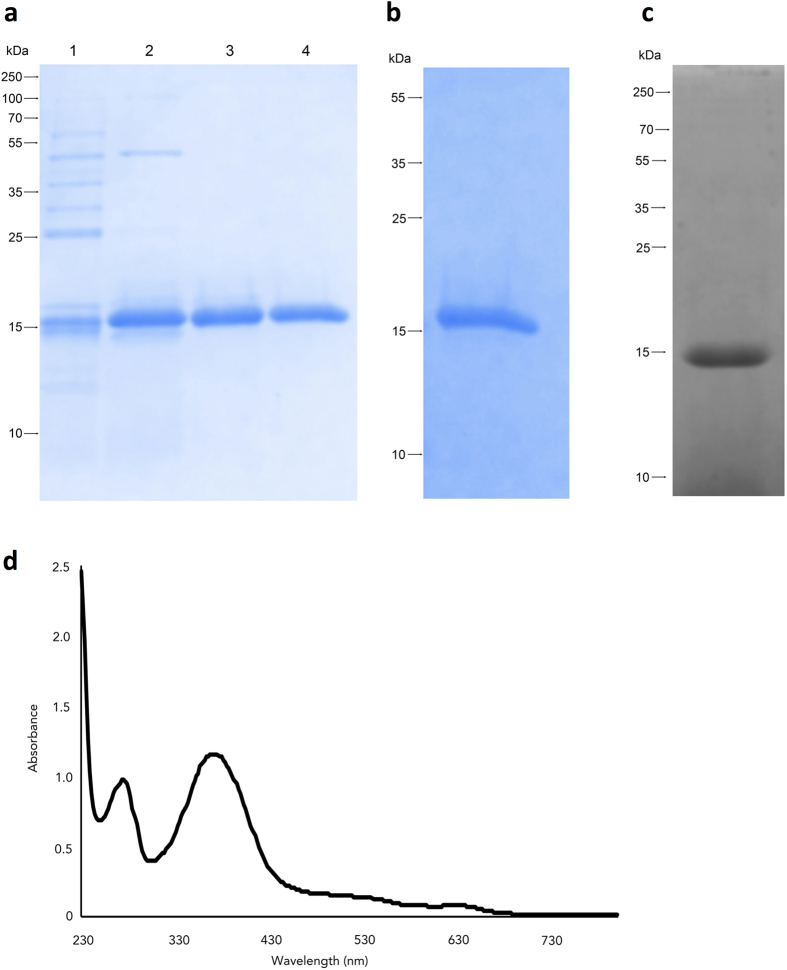
RoxP binds to heme. (**a**) The purification process of RoxP started with the culture supernatant from *P. acnes* strain KPA171202 (1), followed by 50 mM NaCl elution from an anion exchange chromatography (2; HiTrap Q FF pH 8.8), 100 mM NaCl elution from a cation exchange chromatography (3; HiTrap SP FF pH 5.0), and a final 50 kDa MWCO filter purification step (4). (**b**) RoxP was analyzed for its ability to bind to heme. Secreted proteins from *P. acnes* strain KPA171202 were incubated with heme-agarose, eluted, and separated on an SDS-PAGE gel. (**c**) Purified RoxP was stained with *o*-dianisidine to confirm heme binding. (**d**) The interaction of RoxP with heme was further analyzed by measuring the absorbance spectrum of RoxP incubated with heme, displaying a typical spectrum for a heme protein. All experiments were performed at least three times, and are presented as representative figures.

The unique presence of RoxP in *P. acnes* prompted us to investigate its function. Due to the high secretion levels of RoxP (∼10 μg/ml), we decided to purify natively secreted RoxP from the culture medium of *P. acnes* rather than expressing it recombinantly. The purification process was greatly facilitated due to the low molecular weight of the protein (15 kDa), and due to the fact that RoxP is the most abundant protein found in the supernatant of the culture medium of *P. acnes*^22^. By combining ammonium sulphate precipitation, a two-step ion-exchange chromatography (anionic and cationic) and size exclusion we could purify RoxP to approximately 95% homogeneity (Fig. 3a). The identity of the protein was confirmed with mass spectrometry (data not shown). The culture supernatant from 1000 ml bacterial suspension grown to stationary phase generally yielded 3 mg almost pure protein.

### RoxP binds heme

It has long been recognized that *P. acnes* produces and secretes porphyrins^20^,^26^ as byproducts of the vitamin B12 biosynthesis pathway. The high production of porphyrins from *P. acnes* is exploited therapeutically to kill the bacterium^27^. Porphyrin-like molecules are usually used as co-factors in several proteins (such as the methylmalony-CoA mutase). It has not been studied if *P. acnes* secretes proteins containing porphyrins (*e.g*. protoporphyrin IX; further on denoted as heme). To investigate if RoxP could interact with heme, we studied its binding to hemin-agarose. RoxP bound strongly to the column (Fig. 3b), and could also be stained with the heme-specific stain *o*-dianisidine (Fig. 3c). Furthermore, incubation of RoxP with heme and subsequent desalting to remove excess of unbound heme revealed a protein with an absorbance spectrum characteristic for heme proteins with the absorbance peak around 400 nm (Soret band) (Fig. 3d).

### RoxP acts as an antioxidant

**Figure 4:**
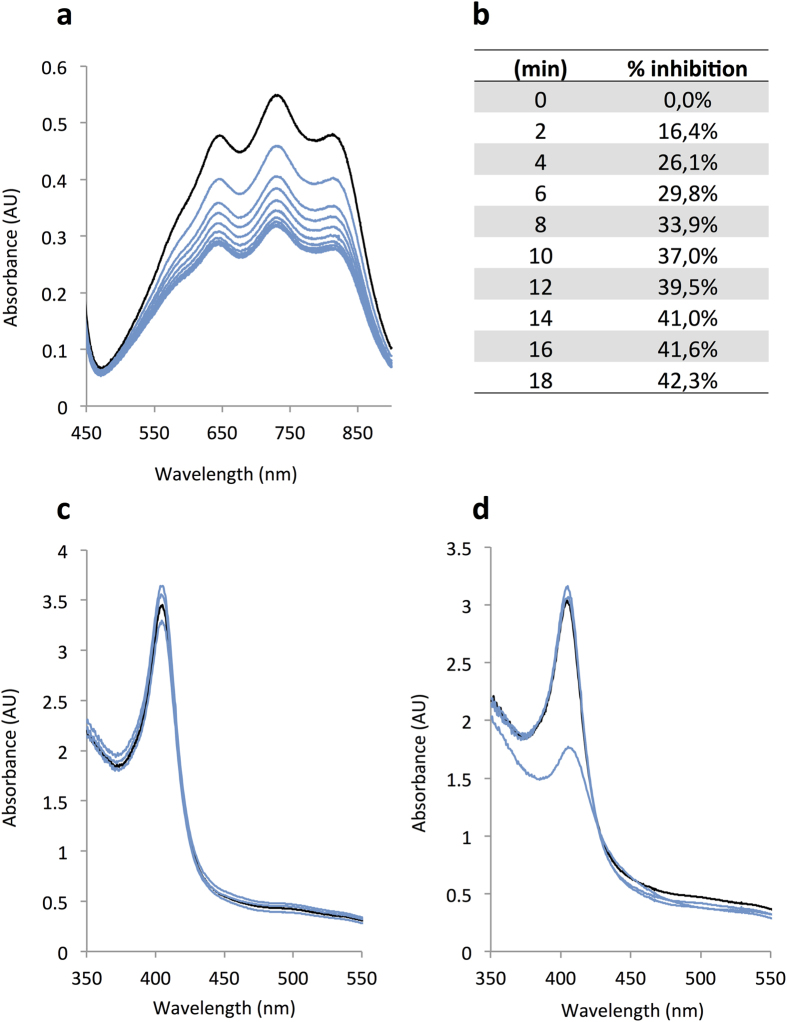
RoxP has antioxidant properties. (**a**) RoxP was incubated with preformed ABTS-radicals and changes in absorbance was measured over time (0–18 min) and summarized as % inhibition (**b**). Culture supernatant from wildtype *P. acnes* (**c**) and Δ*roxP*-mutant *P. acnes* lacking RoxP (**d**) were incubated with hemoglobin and absorbance was measured over time (0–5 h). Black lines represent the starting absorbance, and blue lines changes over time. All samples were analyzed at a maximum absorbance of 0.6 through dilutions, and are reported as measured absorbance multiplied by dilution factor. All experiments were performed at least three times, and are presented as representative figures.

Many heme proteins can modulate the redox status of molecules, being able to reduce oxidized macromolecules^28^. To investigate if RoxP has antioxidant effects, we incubated purified RoxP with preformed ABTS (2,2′-azino-bis(3-ethylbenzothiazoline-6-sulphonic acid)-radicals. Addition of 0.34 μM RoxP was able to reduce the free radicals in a time-dependent manner, reaching a plateau after 12 min (Fig. 4a,b). Increasing concentrations of RoxP resulted in immediate reduction of the absorbance to background levels. PBS served as a negative control, and vitamin E as a positive internal control, with the latter demonstrating a similar degree of antioxidant activity as RoxP (Supplementary Fig. 3).

To better be able to study the biological role of RoxP, we deleted *roxP* from *P. acnes* KPA171202 through mutagenesis according to a recently described protocol^29^. The deletion of *roxP* was verified by PCR amplification and sequencing (data not shown). The successful deletion of *roxP* was further verified by comparing the secreted protein fraction of *P. acnes* KPA171202 (wildtype) and the mutant strain KPA171202Δ*roxP* by 2D gel electrophoresis. The prominent protein spot of RoxP in the 2D gel pattern of the secreted proteins of the wildtype strain was absent in the pattern of the Δ*roxP*-mutant strain (Supplementary Fig. 4).

Next, we investigated the ability of sterile filtered culture supernatants from wildtype and Δ*roxP*-mutant *P. acnes* to protect molecules from oxidation. As a proof of principle to study the antioxidant effect of RoxP, we measured the degradation of oxy-hemoglobin incubated with wildtype or Δ*roxP*-mutant strain-derived supernatants, respectively. Oxidation of hemoglobin results in the degradation thereof, as detected by a decrease of Abs_410 nm_, while putative antioxidant effects of the supernatant will stabilize hemoglobin and limit degradation. The addition of wildtype supernatant resulted in no visible degradation of hemoglobin over a 5 h time period (Fig. 4c). However, addition of the Δ*roxP*-mutant supernatant resulted in complete degradation of hemoglobin within 5 h (Fig. 4d), indicating a reduced ability of the Δ*roxP*-mutant strain to protect the molecule (hemoglobin) from oxidative stress.

### RoxP facilitates *P. acnes* survival in oxic environments *in vitro*

**Figure 5:**
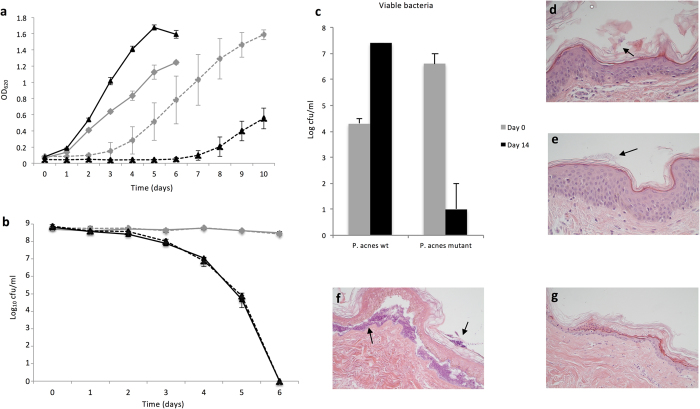
RoxP significantly facilitates the survival and colonization of *P. acnes* in oxic environments. The growth under anoxic (solid lines) and oxic (dashed lines) conditions for *P. acnes* strain KPA171202 (gray) and its isogenic Δ*roxP*-mutant (black) was followed by optical density measurements for six and ten days, respectively (**a**). A stationary phase culture of KPA171202 (gray) and the Δ*roxP*-mutant (black) was washed, and incubated in an oxic environment. To both samples, purified RoxP was added (0.02 mg/ml, dashed lines). Cfu was measured daily through plating of bacteria (**b**). *P. acnes* was added to human skin *ex vivo* from the breast region. Presence of *P. acnes* was measured 2 h and 14 days after inoculation and washing of the cells (n = 6) (**c**). Visual inspection of the skin biopsy using immunohistochemistry at 2 h (**d,e**) and 14 days (**f**,**g**) revealed the presence of wildtype bacteria at both time points (**d**,**f**), colonizing the top of epidermis, in between epidermis and dermis, and partly within epidermis and dermis. For the Δ*roxP*-mutant, presence of bacteria could only be detected 2 h (**e**) after inoculation, but not after 14 days (**g**). Arrows indicate bacteria. All images were recorded at a 40x magnification. All experiments were performed at least with three biological replicates, and are presented as mean ± SD.

Other bacterial antioxidant proteins have been suggested to be of importance for oxygen tolerance^30^. To investigate the importance of RoxP for *P. acnes* survival in an oxic environment, cultures were grown under anaerobic and aerobic conditions. The Δ*roxP*-mutant strain had a slightly faster growth curve as compared to the wildtype during anaerobic conditions (Fig. 5a, solid lines), but had a much slower growth during aerobic conditions (Fig. 5a, dashed lines). Likewise, wildtype *P. acnes* was able to survive in a normal air oxygen environment for an extended period of time, while the Δ*roxP*-mutant was sensitive to oxygen, with no live bacteria present after six days of incubation (Fig. 5b, solid lines). Addition of free RoxP (0.02 mg/ml) enhanced the initial survival of both the wildtype and the Δ*roxP*-mutant strains (27 ± 16%, and 20 ± 22%, respectively), but had no lasting effect (Fig. 5b, dashed lines). Though a trend, the increased initial survival upon RoxP supplementation was not statistically significant.

### RoxP is necessary for long-term *P. acnes* colonization of human skin

To further investigate the biological impact of RoxP for *P. acnes*, we colonized human skin *ex vivo* with wildtype and Δ*roxP*-mutant *P. acnes*. Briefly, skin explants from breast reduction surgery were removed of any subcutaneous fat, and punched to generate small (0.6 cm^2^) pieces of skin. The tissues could be maintained for several weeks in cell media. Bacteria were applied to the epidermal side of each explant. Both strains were able to initially adhere to and colonize the skin (2 h after inoculum) (Fig. 5c–e), suggesting that the presence of RoxP is not involved in initial adherence to skin. However, after prolonged incubation (14 days), only the wildtype strain was significantly present on the skin, having propagated ∼3 log, while the Δ*roxP*-mutant strain was barely detectable, even though demonstrating a higher initial adherence to cells (Fig. 5c,f–g). The presence of erythromycin as a selective pressure did not affect the growth of the Δ*roxP*-mutant *in vitro* (data not shown). Thus, this experiment revealed the crucial role of RoxP for the ability of *P. acnes* to long-term colonize human skin.

## Discussion

Being one of the most common microorganisms on the skin, *P. acnes* has been attributed to be both a commensal and an opportunistic pathogen. Still, most individuals are asymptomatic carriers, with colonizing bacteria that may even benefit their host^10^. Colonizing the skin requires the bacterium to tolerate oxygen and be able to protect itself from ROS. Several enzymes involved in redox reactions, that are able to counteract oxidation, rely on heme as a co-factor for their activity. We therefore investigated if *P. acnes* secreted heme-containing proteins, and identified RoxP as an abundant heme protein. Though heme may not be present in measurable quantities on the skin, it has been reported that *P. acnes* produces several heme-like molecules, *e.g.* porphyrins^26^. The ability of these porphyrins to interact with RoxP and thus serve as an endogenous substrate is currently under investigation.

*P. acnes* has earlier been shown to harbor intracellular catalases and superoxide dismutases, enabling bacteria to cope with cytoplasmic oxidative stress^31^. However, the role of a secreted enzyme is likely different from that of intracellular enzymes. Here, we showed that RoxP is able to both reduce free radicals and protect molecules from oxidation. To the best of our knowledge, this is the first characterized bacterial extracellular enzyme with antioxidant activity. In terms of activity, RoxP is on par with well-studied human antioxidants, including vitamin C, vitamin E and reduced gluthathione^32^, as well as with the plasma protein α_1_-microglobulin shown to have radical scavenging activity^33^. The antioxidant activity of RoxP might thus be beneficial for the bacterium as well as for the host, detoxifying free radicals on skin, suggesting a putative function of RoxP for maintaining the redox homeostasis on the skin.

In many skin diseases, including psoriasis, a significant lower abundance of *P. acnes* at the site of disease as well as for the skin in general has been detected^12^. Several lines of evidence suggest that skin diseases, including atopic dermatitis and psoriasis, are partly mediated by oxidative stress^34^. A dysbiosis in the skin microbiome, with a lowered abundance of *P. acnes* may thus confer a lowered ability to tolerate oxidative stress, thus culminating in a disrupted redox homeostasis. As such, colonization with *P. acnes* on the skin may confer protection against oxidative stress through its secreted protein RoxP, reducing the risk of developing several skin diseases. Further investigation into this area will be needed to confirm this hypothesis.

Regarding the bacterial advantage, RoxP is favorable for *P. acnes* in order to survive and grow in oxygen-rich environments. Being a skin commensal, though preferably in the deeper regions of the skin (*e.g.* sebaceous follicles), it is inevitable that the bacterium will be exposed to oxygen. Thus, to cope with this stress, the bacterium must have evolved systems to handle oxygen and its radical species. For the same reasons, it is to be expected that such a factor would be constitutively and strongly expressed. This is in contrast to an intracellular heme oxygenase from *Clostridium tetani*, also suggested to be involved in oxygen tolerance, that preferentially is expressed in stationary phase^30^. Our data support the importance of RoxP for oxygen tolerance of *P. acnes*, enabling both growth and survival in an oxic environment. Interestingly, the isogenic Δ*roxP*-mutant displays an increased growth rate in anaerobic conditions, as compared to the wildtype. This may reflect the burden of the high production of RoxP in the wildtype, a protein of less importance in an anoxic environment. However, in an oxic environment, RoxP facilitates skin colonization of *P. acnes*. Since only the strain KPA171202 (type IB) was used, the results of our study are limited to this particular strain. This is the only strain of *P. acnes* that has so far been successfully genetically manipulated. The used strain, however, has the most common *roxP* genotype, that is shared in type IA and IB strains as well as some type II strains. Thus, the results have likely a broader relevance. Still, we cannot rule out that other strains of *P. acnes* may differ in terms of their growth properties under oxic environments.

A constitutive expression of *roxP* is likely a necessity, since single addition of exogenous RoxP to *P. acnes* in an oxic environment only momentarily affected the viability of *P. acnes*, but had no significant long lasting effect. The reason for the lack of functionality of exogenous RoxP needs to be further investigated. It is possible that another factor might be necessary for full RoxP activity, or that the partial purification of RoxP has affected *in vitro* functionality.

Due to its ability to interact with heme, it is possible that RoxP can also interact with the porphyrins produced by *P. acnes* itself. How such an interaction will affect the production, and functionality of RoxP remains to be investigated, but may be a limiting factor for the activity of exogenously added RoxP *in vivo*.

RoxP has several evolutionary interesting features, being the most abundantly secreted protein from *P. acnes*^22^, unique for *P. acnes* and the tentatively named species *P. humerusii*, as well as highly conserved within these species. This suggests that RoxP is of importance for their specific habitat, and might thus be of importance for the host-bacterium interaction.

The constitutive expression of RoxP distinguishes it from most common host-interacting factors, which often display growth phase-dependent expression^35^. The importance of secreted RoxP for the interplay between the RoxP-producing strain and its local environment is intriguing. Several other facultative anaerobes, including the common skin bacterium *Finegoldia magna* inhabits the skin^36^, but it has not been studied whether these bacteria have a symbiotic interaction, benefiting each other’s survival. Further studies will be needed to elucidate the putative symbiosis between skin bacteria.

Commensal bacteria on the skin are generally regarded to be beneficial for the host, but few mechanisms have been shown to support this notion. Here we have characterized a unique secreted antioxidant enzyme derived from *P. acnes* that protects the bacterium from oxidative damage, enables its survival in an oxic environment, and possibly assists in maintaining a healthy skin redox homeostasis. Further studies will elucidate the importance of RoxP for enabling symbiotic interactions between *P. acnes* and its human host.

## Methods

### Bacterial strains and growth conditions

*P. acnes* isolates were cultivated from −80 °C stock solutions in Tryptic Soy Broth (TSB; Bacto, Mt Pritchard, NSW, Australia) under strict anaerobic conditions for 3–7 days, until reaching stationary phase. Additional information about the *P. acnes* isolates used can be found elsewhere^5^.

For oxygen-sensitivity experiments, *P. acnes* strain KPA171202 and the isogenic Δ*roxP*-mutant were cultured in TSB under strict anaerobic conditions (anaerobic workstation) until reaching stationary phase. All media were pre-reduced anaerobically for 24 hours, before addition of bacteria. Bacteria were diluted 1:100 in non-reduced TSB, and incubated at 37 °C in an oxygen atmosphere. Optical density at 620 nm was measured every 24 hours. Likewise, stationary phase bacteria were washed in PBS, and resuspended to a concentration of ∼5 × 10^8^ colony forming units (cfu)/ml. PBS or RoxP (0.02 mg/ml) was added, and samples were allowed to incubate at room temperature in an air oxygen-saturated environment. Samples were plated every 24 hours in order to count cfu.

### Sequencing of *roxP*

The gene *roxP* was PCR-amplified in isolates AD9, AD20, AD24, AD26, AS4, AS8, AS23, AS26 and AS39 using primer roxP_for and roxP_rev (Table 1). The PCR product was gel purified (Qiagen) and ligated to the pCR2.1 vector (Invitrogen) according to manufacturer’s instruction, before being transformed into *E. coli* TOP10 cells. Plasmids were purified and sequenced using GATC Lightrun.

### *roxP* expression in *P. acnes*

*P. acnes* mRNA was isolated using RNeasy Mini Kit (Qiagen) supplemented with Bacteria Protect Reagent (Qiagen). Briefly, 1 ml *P. acnes* culture was pelleted, resuspended in a mixture of PBS and Bacteria Protect Reagent and incubated for 5 min. Bacteria were lysed in TE-buffer (10 mM Tris-HCl pH 8.0, 1 mM EDTA) containing 15 mg/ml lysozyme (Sigma-Aldrich) and proteinase K (Qiagen); incubating for 10 min with agitation. Reagent RLT (RNeasy kit) was added and particles removed by centrifugation. The remaining supernatant was mixed with ethanol, and the sample added to RNeasy spin columns according to manufacturer’s instructions.

Gene expression of *roxP* and *gapdh* was analyzed by quantitative real-time PCR (qPCR) using Power SYBR Green RNA-to-Ct 1-step Master Mix (ThermoFisher), 100 nM of primers (gapdh_for/rev, qRT-PCR_roxP_for/rev) (Table 1) together with 5–10 ng mRNA. Samples were run on an iCycler iQ (Bio-Rad) with reverse transcription at 48 °C for 30 min, followed by 95 °C for 10 min and 40 cycles with 15 s at 95 °C and 60 °C for 1 min. Gene expression levels were quantified using the ΔΔCt-method.

### RNA preparation and RNA-sequencing

Total RNA from *P. acnes* (KPA171202 (type IB), and 12.1.L1 (type IA)) grown under anaerobic conditions to the exponential and stationary growth phase in BHI medium at 37 °C was prepared with the RNA PowerSoil Total RNA Isolation Kit (MoBio, USA), following manufacturer’s instructions. RNA quality was controlled using the Bioanalyzer (Agilen Technologies, USA). The cDNA libraries were constructed by Vertis Biotechnology AG, Germany, as previously described^37^,^38^. The cDNA libraries were sequenced using a HiSeq 2000 instrument (Illumina) in a single-read mode and 100 cycles. Detailed description of procedures used for quality control, read mapping, expression graph construction and normalization of expression graphs have been published previously^37^. For graph visualization the Integrative Genome Browser (IGB v8.5.4) was used^39^.

### Phylogenetic analysis

A phylogenetic tree based on the extracted RoxP protein sequences of all *P. acnes* and *P. humerusii* genomes available in GenBank was generated using the minimum-evolution algorithm according to the maximum composite likelihood model in the software program MEGA 5.2^40^, and validated by bootstrap analysis based on 500 replications.

### Mutagenesis of *P. acnes roxP*

The RoxP knockout mutant was generated as described previously^29^. *P. acnes* strain KPA171202 was used as the wild-type strain and *Escherichia coli* DH5α was used as a recipient in all cloning steps, unless stated otherwise. In brief, a 500 bp region upstream and a 500 bp region of the 3′ end of the gene PPA1939 (*roxP*) were amplified using the primer combinations PPA1939_1/PPA1939_2 and PPA1939_3/PPA1939_4 (Table 1), respectively. The fragments were gel-purified, digested with Acc65I and ligated. After column purification of the ligation mixture (QIAquick, Qiagen), a PCR was carried out using the ligation mixture as a template with the primer combination PPA1939_1/PPA1939_4. The 1 kb product was gel-purified and cloned into pGEM-T-Easy. Positive *E. coli* DH5α clones were obtained and isolated plasmids were tested for the correct insert by restriction digestion. Plasmid DNA was subsequently digested with Acc65I and ligated with the Acc65I-digested fragment containing the erythromycin-resistance cassette; this fragment was amplified using genomic DNA of *Saccharopolyspora erythraea* (DSM no. 40517) as a template using the primers ermE_for and ermE_rev (Table 1). The ligation mixture was transformed into *E. coli* DH5α. Ampicillin (100 μg/ml)-resistant clones were obtained. Prior to electroporation into *P. acnes* KPA171202, plasmid DNA was extracted from positive clones and transformed into the dam-negative *E. coli* strain GM2199.

Competent cells of *P. acnes* KPA171202 were prepared as described previously^29^. Electrocompetent *P. acnes* cells were freshly prepared before the electroporation, which was carried out using a GenePulser (BioRad), and 0.1 cm electroporation cuvettes. Plasmid DNA (6 μg; isolated from the dam-negative *E. coli* strain GM2199) was added to 50 μl competent *P. acnes* cells. GenePulser settings of 50 μF, 400 Ω and 1.5 kV were used. Subsequently, cells were recovered in 1 ml pre-warmed BHI medium, and centrifuged (5 min, 3000 g). The resulting pellet was resuspended in 100 μl BHI broth and plated out on a Brucella agar plate without any antibiotics. The plate was incubated for 24 h at 37 °C under anaerobic conditions. On the following day, bacteria were harvested using a sterile cotton swab, resuspended and washed in pre-warmed BHI broth, and re-plated on Brucella agar plates containing 10 μg/ml erythromycin, and incubated for 4–6 days. All obtained clones were tested by PCR to validate the correct knock-out of PPA1939.

### Purification of RoxP

Stationary phase *P. acnes* isolate KPA171202 were collected by centrifugation at 3600 g for 10 min. The culture supernatant was sterile filtered (0.22 μm) and precipitated with 50% ammonium sulfate. The pellet was resolved in 10 mM Tris-HCl pH 8.8 and dialyzed overnight (MWCO 3,500). The sample was loaded on an equilibrated HiTrap Q FF column (GE Healthcare, Uppsala, Sweden), and eluted with 50 mM NaCl (10 mM Tris-HCl pH 8.8). A second dialysis was performed (50 mM NaOAc-HOAc pH 5.0), samples were run on a HiTrap SP FF column (GE Healthcare), and eluted with 100 mM NaCl (50 mM NaOAc-HOAc pH 5.0). Finally, fractions were run through a 50 kDa MWCO-filter (Amicon Ultra, Ultracel-50K). The homogeneity of RoxP was verified using SDS-PAGE gels.

### MALDI-TOF analysis

Culture supernatant from stationary phase *P. acnes* isolate KPA171202 was precipitated with 0.2% v/v HCl in acetone. The proteins were pelleted (10,000 g, 30 min, 4 °C) and supernatant discarded. The pellet was dissolved in 0.1 M Tris-HCl pH 7.5, 0.1 M NaCl, and the proteins were separated on a 12% SDS-PAGE^41^, and stained with 0.25% Coomassie Brilliant Blue. Bands were cut out using a sterile scalpel and analyzed using MALDI-TOF MS/MS as described elsewhere^42^. Briefly, the gel spots were destained with 50% acetonitrile (ACN) in 25 mM NH_4_HCO_3_, and reduced and alkylated using DTT and iodoacetamide, respectively. The gel spots were washed with NH_4_HCO_3_, dehydrated with ACN, and dried using a speed vacuum. The gel spots were digested with trypsin (Promega) over night at 37 °C. Peptides were eluted with the addition of 5% trifluoroacetic acid in 75% ACN. Eluted peptides (1 μl) were spotted on a target plate before a matrix of 2 mg/ml alpha-cyano-4-hydroxycinnamic acid was added. Additionally 1 μl of peptides was added in order to increase the signal. The analysis was performed on an ESI-TRAP (Waters), using a mass range m/z from 50–1800. Only spectra from ions with charge states 2 and 3 were acquired.

### Hemin agarose purification

Hemin agarose suspension (1:1) (Sigma-Aldrich) was washed with 20 mM Tris-HCl pH 7.5, 0.15 M NaCl 3 × 5 min. RoxP from *P. acnes* isolate KPA171202 (15 μl) was added to 100 μl hemin agarose, incubated for 2 h at room temperature and washed 5 × 500 μl with Tris-HCl pH 7.5. The sample was finally pelleted (8,000 g 2 min) and resuspended in 30 μl Nu-PAGE sample buffer (Invitrogen) and reducing agent (Invitrogen). The samples were incubated for 10 min at 70 °C, pelleted and applied to the 4–12% gradient gel.

### Electrophoresis and *o*-dianisidine staining

SDS-PAGE was performed using reducing conditions as described by Laemmli^41^. For heme-staining, Nu-PAGE gels, 4–12% (Invitrogen) were used. Samples were incubated for 30 min at 20 °C with sample buffer containing 0.25 M Tris-HCl pH 6.8, bromophenol blue, 2% SDS and 25 mM DTT (final concentration). The gels were pre-run for 1 h before loading of the samples. After electrophoresis (100 V) the gels were stained with Coomassie Blue G-250 for detection of proteins or 6 mM ortho-dianisidine-HCl (*o*-dianisidine-HCl) and 18 mM H_2_O_2_ for detection of heme. After electrophoresis the gels were washed in 10% TCA for 10 min, washed twice with H_2_O for 10 min, and finally incubated in 20 ml stain containing 2 ml 0.5 M Na-citrate, pH 4.4, 6 mM *o*-dianisidine-HCl and 0.4 ml H_2_O_2_. The staining was stopped after 30 min and kept in water.

### Binding of heme to RoxP

Heme (0.5 mM in DMSO) was incubated with 15 μg RoxP, desalted on pre-equilibrated PD10 columns (GE Healthcare, Sweden) and the top fractions used for analysis.

### 2D protein gel analysis of secreted proteins

This method has been previously described^22^. In brief, wildtype and mutant strains were cultured at 37 °C on Brucella agar plates under anaerobic conditions for three days. Plate-grown bacteria were resuspended and washed in BHI broth. Twenty ml BHI broth was inoculated with *P. acnes* (OD_600_ 0.01) and grown for 12–72 h at 37 °C and 160 rpm in an anaerobic jar. After 14–18 h, the cultures typically reached the mid-exponential growth phase with an OD_600_ of 0.5–0.6. The exponential cultures were centrifugated for 15 min at 20,000 g, 4 °C, and the supernatant was filtered through a 0.22 μm filter to remove residual bacteria. Extracellular proteins were precipitated using a modified trichloroacetic acid (TCA) method as described previously^43^. In brief, the filtrate (100 ml) was mixed with 100% TCA to a final concentration of 10% and incubated overnight at 4 °C. The mixture was centrifugated for 30 min (20,000 g, 4 °C) and the resulting pellet resuspended in 100 ml of acetone and dissolved using an ultrasonic water bath. The mixture was centrifugated as before, washed twice with acetone, and the resulting pellet air dried. For two-dimensional gel electrophoresis, protein samples were solubilized for 30 min at ambient temperature in 9 M urea-1% 3-[(3-cholamidopropyl)-dimethylammonia]-1-propanesulfonate (CHAPS)-70 mM dithiotheitol (DTT)-2% Servalyte 2–4 (Serva). Protein species were separated by a small-gel-2-DE system^44^. The samples containing 200 μg of protein were applied to the anodic side of the isoelectric focusing gel containing ampholytes in the pH range 2–11. The SDS-PAGE of the second dimension was performed using 15% acrylamide gels (7 cm × 8 cm). Protein spots were visualized by staining with Coomassie Brilliant Blue G-250.

### Antioxidant effect on ABTS-radicals

2,2′-azinobis(3-ethylbenzothiazoline-6-sulfonic acid) diammonium salt (ABTS) was dissolved in ddH_2_O to a concentration of 7 mM. Production of ABTS radical cations were performed overnight at RT by mixing ABTS (0.4 mM final concentration) with potassium persulfate (2.45 mM final concentration), kept dark. Prior to usage, ABTS radical solution was dissolved in PBS to an absorbance of ca 0.7 at 734 nm. To diluted ABTS radical solution (90 μl), 10 μl antioxidant solution (final concentration 0.34–15 μM) was added, and the absorbance spectrum at 350–950 nm was recorded. Vitamin E was used as a positive control, and buffer alone as a negative control.

### Degradation of oxy-hemoglobin

Oxy-hemoglobin used for incubation with RoxP was purified as described elsewhere^45^ using DEAE Sephadex ion-exchange of erythrocyte hemolysates chromatography according to Winterbourn^46^. Hemoglobin (80 μg/ml final concentration) was added to bacterial supernatants, and incubated at 37 °C, shaking, for 0–5 h. Absorbance spectra at 200–1200 nm were recorded.

### Spectrophotometric methods

Absorbance spectra was measured on a Beckman DU 640i spectrophotometer (Beckman Instruments, CA, US) using a scan rate of 1200 nm/min and protein concentration 10–40 μM. Spectrophotometric studies were performed by recording the absorbance with 1–5 min intervals for up to 1 hour on a spectrophotometer in the UV-VIS region between 300 and 800 nm.

### *Ex vivo* colonization of *P. acnes*

Subcutaneous fat was excised and removed from human skin from a breast reduction. Skin pieces were punched (0.6 cm^2^), and a silicon sealant (Mepiseal) was applied around the edge of each punched explant, to better contain the bacteria on the epidermis. The tissues were placed in Netwells that were transferred to 12 well plates and incubated in DMEM supplemented with 10% FBS, 1% glutamax, and 10 μg/ml erythromycin for the Δ*roxP*-mutant, at 37 °C, 10% CO_2_ and 95% humidity. Stationary phase *P. acnes* (wildtype and Δ*roxP*-mutant strains) were added in the center on the epidermal side of each biopsy (10  μl, 2 × 10^9 ^cfu/ml; DMEM as control). The inoculums were allowed to dry in, and colonize the skin during 2 h at 37 °C. The skin explant was cultured for two weeks, with daily changes of nutrient. Samples were taken for analysis at 2 h after inoculation, and 14 days after inoculation. Biopsies were gently washed in PBS to remove any loosely adherent bacteria, cut into smaller sizes (*e.g.* 10 pieces per biopsy) and homogenized by vortexing the samples together with glass beads for 2 min at maximum speed. Serial dilutions were made in PBS, and plated in duplicates, incubating anaerobically for up to 11 days before analyzing growth of bacteria. For histological analyses, skin pieces were incubated in 5 ml formaldehyde, dehydrated and embedded in paraffin, cut into 5 μm sections, deparaffinized, rehydrated and stained with hematoxylin and eosin. All experiments related to the *ex vivo* study was conducted by the company Medibiome. No ethical approval was necessary for the work with residual tissues.

### Statistical analysis

All analyses were calculated with the non-parametric Fisher’s exact test. Only a p-value less than 0.05 was considered statistically significant.

## Additional Information

**Accession codes:** The GenBank accession numbers for the *roxP* genes are for AD9 (JN051665), AD20 (JN051666), AD24 (JN051667), AD26 (JN051668), AS4 (JN051669), AS8 (JN051670), AS23 (JN051671), AS26 (JN051672) and AS39 (JN051673).

**How to cite this article**: Allhorn, M. *et al*. A novel enzyme with antioxidant capacity produced by the ubiquitous skin colonizer *Propionibacterium acnes. Sci. Rep.* **6**, 36412; doi: 10.1038/srep36412 (2016).

**Publisher’s note**: Springer Nature remains neutral with regard to jurisdictional claims in published maps and institutional affiliations.

## Supplementary Material

Supplementary Information

## Figures and Tables

**Table 1:** Primers used in this study.

Primer	Sequence (5′–3′)
PPA1939_1	CGTCGACACACCATGCCGAA
PPA1939_2	gcggtaccCCGATGTGTCGTACGTATGG
PPA1939_3	gcggtaccCGATGAGAGCCAACTTCCAG
PPA1939_4	TCATCGCTATGAGCGCCAAC
ermE_for	GAGCGCACCGACCCGGTCGT
ermE_rev	TGCTGCGCCAGCGTTGTGCG
roxP_for	CATCAGGTTCACGAAACCGAAA
roxP_rev	AACGATCACGGTGGCAGGATA
qRT-PCR_roxP_for	GCATCTAGCCCTCTCACCAT
qRT-PCR_roxP_rev	CTGAGAGTCCGGTAGGTGGT
gapdh_for	GCATCATGACTACCGTCCAC
gapdh_rev	CGGTGGTCTCCTTAGAGGTC
