# Supplementary material for: A novel enzyme with antioxidant capacity produced by the ubiquitous skin colonizer Propionibacterium acnes
Source: Sci Rep. 2016 Nov 2;6:36412. doi: 10.1038/srep36412 (PMC5090349; doi:10.1038/srep36412)
Supplement: Supplementary Information [file srep36412-s1.pdf]

A novel enzyme with antioxidant capacity produced by the ubiquitous skin colonizer *Propionibacterium acnes*

Maria Allhorn[1], Sabine Arve[1], Holger Brüggemann[2], Rolf Lood[1*]

Supplementary Information

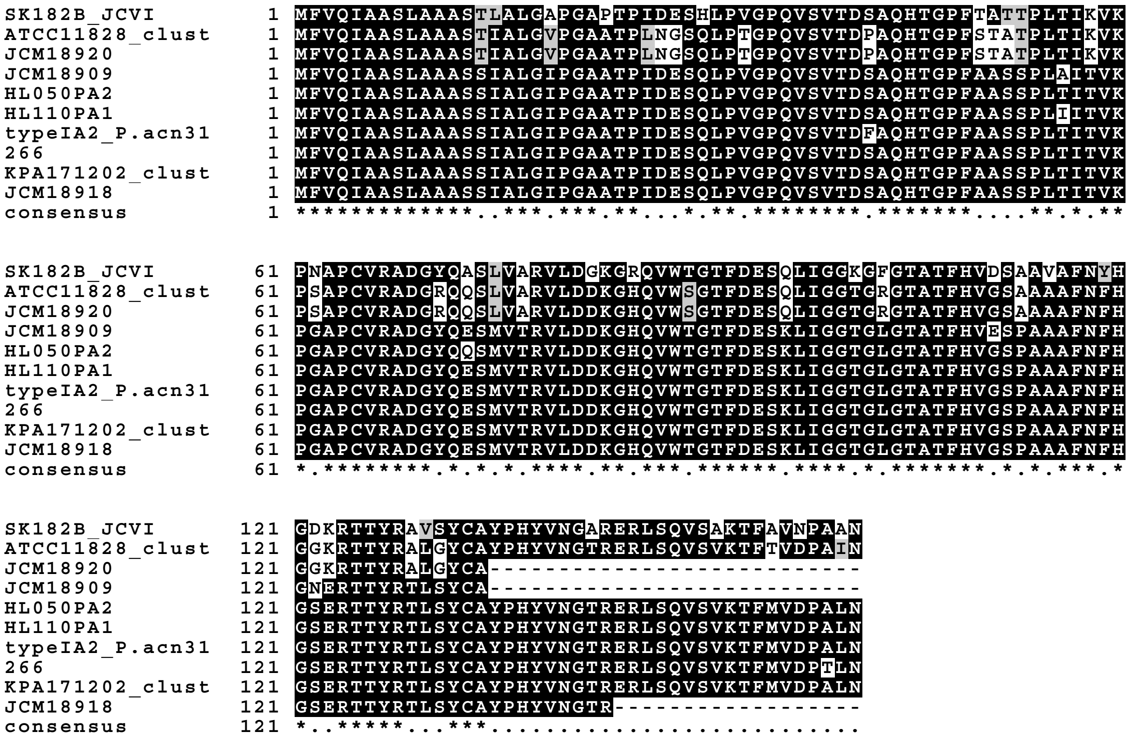

Supplementary Fig. 1. Alignment of RoxP homologs of *P. acnes*. The common RoxP homologs were extracted from *P. acnes* genomes and an alignment was calculated with Clustal Omega and visualized with BoxShade. The most common homolog is represented by strain KPA171202; 58 strains carry an identical copy (see Fig. 1). Most other homologs (represented here by strain 266, HL050PA2, HL110PA1, type1A2_P.acn31) share 99% protein identity. A cluster of 11 strains (represented in this figure by strain ATCC11828) has an identity of 83% on protein level. Furthermore, some strains apparently carry a truncated RoxP (JCM strains).

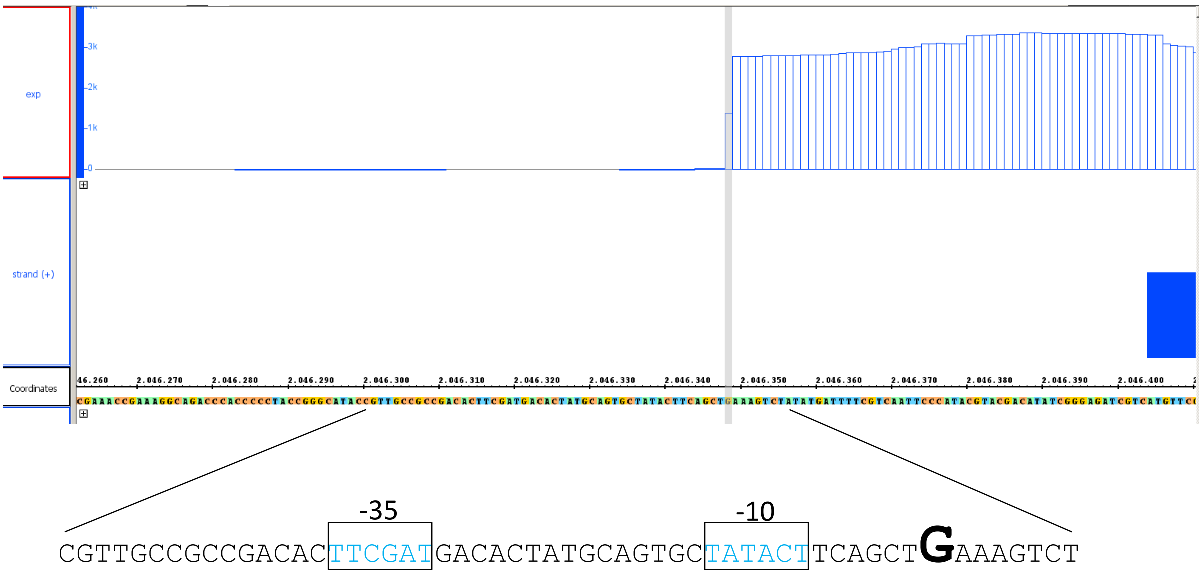

Supplementary Fig. 2A. Upstream region of *roxP*: Identification of the transcriptional start site and the promoter region. The transcriptional start site is highlighted (‘G’) as well as the predicted promoter region.

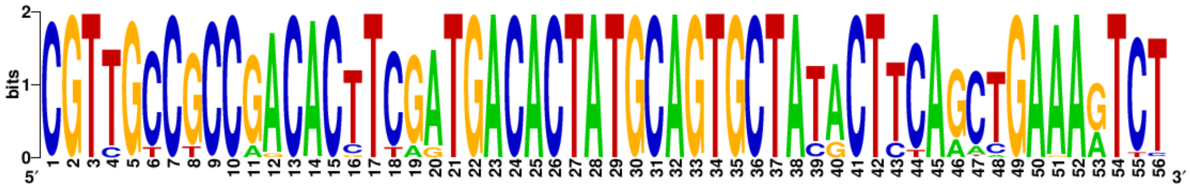

Supplementary Fig. 2B. Consensus sequence of the *roxP* upstream region based on an alignment of sequences from 94 strains. The alignment was created with MEGA, and the consensus sequence was visualized with WebLogo.

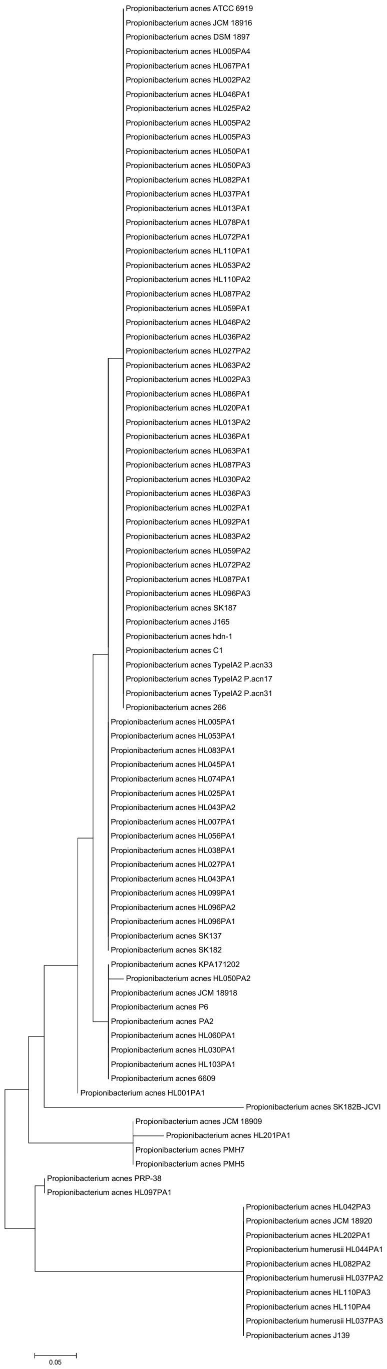

Supplementary Fig. 2C. Phylogenetic analysis of *roxP* upstream region. The *roxP* upstream region, containing the transcriptional start site and the predicted promoter (shown in Fig 2SA), was taken and blasted against all *P. acnes* genomes. The corresponding homologous sequences were extracted and phylogenetically analyzed with MEGA6.

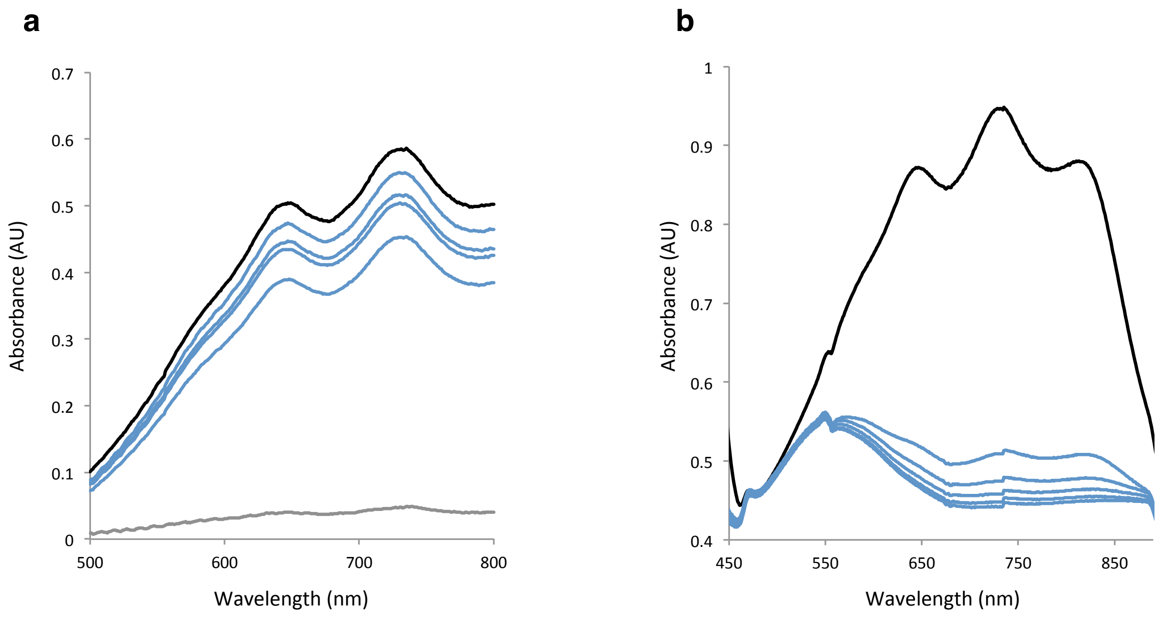

Supplementary Fig. 3. Vitamin E and RoxP reduce preformed ABTS-radicals. (A) Vitamin E (black line: control; gray line: 15 μM vitamin E; blue lines: 1.5 μM vitamin E over 0-5 min) was incubated with preformed ABTS-radicals and changes in absorbance was measured over time (0-5 min). (B) RoxP (black line: control; blue lines: 34 μM RoxP over 10-60 min) was incubated with preformed ABTS-radicals and changes in absorbance was measured over time (10-60 min).

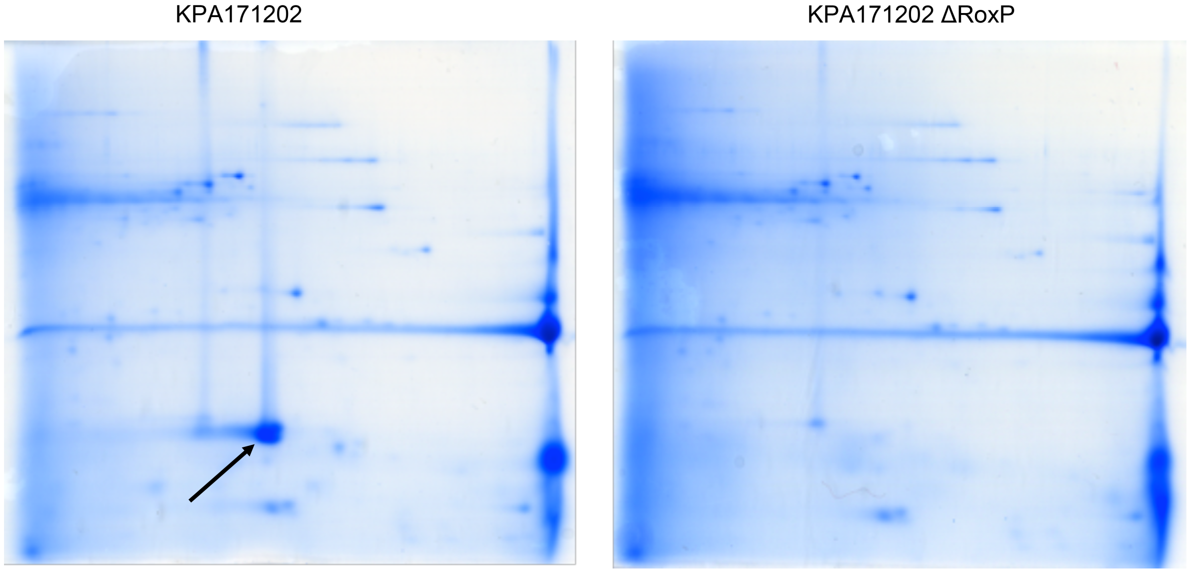

Supplementary Fig. 4. Confirmation of successful deletion of *roxP* by 2D gel electrophoresis of secreted proteins of *P. acnes*. RoxP was previously identified among the abundantly secreted proteins. 2D protein gel electrophoresis of secreted proteins of the wildtype and Δ*roxP*-mutant strains clearly showed that RoxP (arrow) is absent among the secreted proteins of the Δ*roxP*-mutant. This confirms that the *roxP* gene was successfully knocked out.
